# Theoretical and experimental investigation of the impact of oil functional groups on the performance of smart water in clay-rich sandstones

**DOI:** 10.1038/s41598-024-71237-1

**Published:** 2024-08-30

**Authors:** Alireza Kazemi, Saeed Khezerloo-ye Aghdam, Mohammad Ahmadi

**Affiliations:** 1 Department of Petroleum and Chemical Engineering, College of Engineering, Sultan Qaboos University, P. O. Box 123, Muscat, Oman; 2 Department of Petroleum Engineering, Amirkabir University of Technology (AUT), Tehran, Iran

**Keywords:** Low salinity water, Fine migration, Interparticle forces, Wettability, Acid number, Recovery factor, Energy science and technology, Engineering

## Abstract

This research investigated the effect of ion concentration on the performance of low salinity water under different conditions. First, the effect of injection water composition on interparticle forces in quartz-kaolinite, kaolinite-kaolinite, and quartz-oil complexes was tested and modeled. The study used two oil samples, one with a high total acid number (TAN) and the other with a low TAN. The results illustrated that reducing the concentration of divalent ions to 10 mM resulted in the electric double layer (EDL) around the clay and quartz particles and the high TAN oil droplets, expanding and intensifying the repulsive forces. Next, the study investigated the effect of injection water composition and formation oil type on wettability and oil/water interfacial tension (IFT). The results were consistent with the modeling of interparticle forces. Reducing the divalent cation concentration to 10 mM led to IFT reduction and wettability alteration in high TAN oil, but low TAN oil reacted less to this change, with the contact angle and IFT remaining almost constant. Sandpack flooding experiments demonstrated that reducing the concentration of divalent cations incremented the recovery factor (RF) in the presence of high TAN oil. However, the RF increment was minimal for the low TAN oil sample. Finally, different low salinity water scenarios were injected into sandpacks containing migrating fines. By comparing the results of high TAN oil and low TAN oil samples, the study observed that fine migration was more effective than wettability alteration and IFT reduction mechanisms for increasing the RF of sandstone reservoirs.

## Introduction

Using low salinity water is a challenging method for enhanced oil recovery^1–3^. The mechanism that comprehensively explains the function of this approach has not been proposed^4,5^. Low salinity water flooding alters the rock porous media substantially^6–8^, which can result from different interactions like rock/fluid interplay, fluid/fluid interplay, and rock/rock interplay^9^. Therefore, investigating and identifying the strength of this approach in extended situations is essential. The pivotal mechanism of this approach to rock inner structure is challenging^10,11^. These mechanisms can either upgrade or worsen the EOR approach^12^.

Recent papers have identified several impactful mechanisms such as altering the rock wettability, Migration of fine particles, lowering oil/water IFT, pH, and salt-in effects^6,13,14^. Alteration of wettability and migration of fine particles have more effect than other mechanisms^11^. However, numerous studies have already demonstrated that altering rock wettability does not always happen^15^. This mechanism relies on the reservoir rock and fluid characteristics^15^. There are cases in which implementing low-salinity water can worsen the condition of wettability, particularly in reservoirs with carbonate lithology. Additionally, the fine migration mechanism is controversial, as researchers do not fully agree on its effectiveness^13^.

In the field of petroleum engineering, the migration of fine particles has been introduced deteriorating and potentially harmful phenomenon in production and injection wells, injection wells, which is an essential topic for researchers^16–19^. Various approaches to deal with this problem have already been suggested, including adjusting the injection rate in injection wells as well as the production rate in production wells^20^, using clay inhibitors^21^ and surfactants^22,23^ to stop the occurrence of this phenomenon. Fine particles are stable in the porous media in a normal situation where the production rate is low and stable^24^. However, applying IOR and EOR approaches may impact the reservoir adversely^25^. Under the implementation of EOR and with changes in injection or production conditions, as well as the reservoir fluid compositions, fines can become suspended and separated from the facing of the pores^26,27^. Drag forces from fluid flow move the fine particles that detach the porous media. This continues unless the fine particles trap behind a narrower pore throat. Therefore, the connection level between pores is reduced, which lowers the formation permeability^28–30^.

Contrary to the findings of many papers, some researchers claim that fine migration is an important mechanism^25,26^. Tor Austad states that clay particles are mandatory for performing the low salinity water approach^26^. The migration of fine particles may raise the recovery factor through two mechanisms. The first one is related to the pore bodies sweeping. When clay particles move forward in the porous media, the oil is expended from the pore bodies^31,32^. The second mechanism is related to the conformance control. The pore throats that have been affected by the injected low salinity water becomes plugged. Thus, water can enter the areas that have not been swept^33–35^.

Another important mechanism is IFT reduction, which has also been identified in some research as a mechanism for improving RF^13,36–38^. However, it is essential to note that this mechanism does not occur in all cases, and sometimes, the IFT between oil and water is entirely indifferent to water salinity^38–40^. In other cases, it is classified as having lower importance than wettability alteration and fine migration^41^. Indeed, it is crucial to recognize that each of these mechanisms occurs under specific conditions. Thus, some low salinity water projects have been unsuccessful due to a lack of attention to the specific conditions required to successfully implement these mechanisms^6,42^. Exploring the science behind phenomena is crucial to recognizing and controlling the impactful factors. This understanding can help researchers design low salinity water formulations tailored to each reservoir's specific characteristics, leading to a more effective and successful EOR approach.

So far, there have been papers in the field of fine migration, as well as a qualitative study of the effect of interparticle forces. However, this study, for the first time, uses these forces to quantify the composition of injected fluids, in which the concentration and types of ions are changing to investigate their effect on the possibility of fine migration, both theoretically and experimentally. It also examines the effect of functional groups in oil on the recovery enhancement mechanism from the point of view of interparticle forces both in the laboratory and in theory. The study used the plate and sphere model and assumed that quartz particles are plates, water denotes the fluid, clay particles, and oil droplets are spheres. The study then performed IFT and wettability measurements to identify the impact of interparticle force on these phenomena. Additionally, several sandpacks with and without clay were made to observe the effect of interparticle force on the migration of fine particles. The study injected various scenarios to investigate the effect of interparticle force on the migration of fine particles and the impact of fine migration on the oil recovery factor. Ultimately, the study aimed to understand the factors that activate and control mechanisms at the particle scale and investigate their contribution to improving RF.

## Methodology

### Theoretical aspect

This area illustrates up to examine the impact of the encompassing water composition on interparticle powers. The DLVO hypothesis was, to begin with, presented for steadiness calculations of colloidal liquids, and it can be utilized to recognize the sort of overwhelming drive between particles^43^. That is rectified. The overall constraint calculated by the DLVO hypothesis decides the attraction and repugnance between two particles. On the off chance that the entire drive is attractive, the particles will tend to total and shape more giant clusters, while on the off chance that the overall constrain is terrible, the particles will tend to scatter and stay isolated^44^.

Researchers illustrated that the attraction force within rock porous media and oil particles may lead to the injection water being released from pores. As a result of the attraction force, most of the oil is adsorbed to the facing of the rock. In contrast, by flooding water, oil with repulsive forces on the facing of the matrix is peeled off from the rock surface. The first case is likely to occur in reservoir conditions. Thus, an effective increment in the recovery factor may be achieved by altering the injected water composition to change this force into repulsion. It is also evident that migrating fine particles exist in most sandstones, and changing the magnitude and size of interparticle force impacts the movement of the particles. Fine migration may damage the rock media and lower the permeability of the rock. Furthermore, migrating fines may also move oil droplets forward in the porous media, enhancing oil recovery. Thus, investigating interparticle forces in various complexs is necessary.

Generally, DLVO theory models the energy of interplay between particles based on the Lifshitz-van der Waals (ULV) and electrostatic (UEL) energies^45^.

1 $$ {U_T} = {U_{LV}} + {U_{EL}}  $$

The interplay of van der Waals is a microscopic force that results in particles being attracted to one another by their mass. The sphere and planar model has been used to calculate the amount of this force. The Van der Waals energy value shall be calculated using a Hamaker approximation method for short distances. Hamaker is needed to compute the ULV interplay strength of the facing particles, which depends on the dielectric features of the particles^45^.

2 $$  {U_{LV}} = \frac{{{A_{132}}r}}{6h} $$

where A_132_ is the Hamaker constant, r denotes the particle radius, and H is the distance between the sphere and plane.

When two particles with EDL come closer, they impose electrostatic forces on each other, which depend on various parameters. The electrostatic energy of particles depends on the distance between them. The below equation calculates the electrostatic interaction energy^45^.

3 $$  {U_{EL}}(h) = \frac{{\pi \varepsilon {\varepsilon_0}r}}{{{K_B}T}}\left[ {2{\zeta_1}{\zeta_2}\ln \left( {\frac{{1 + {e^{ - kh}}}}{{1 - {e^{ - kh}}}}} \right) + (\zeta_1^2 + \zeta_2^2)\ln (1 - {e^{ - 2kh}})} \right] $$

where, $\varepsilon$ denotes the electrolyte's dielectric constant (F/m), ${\varepsilon_0}$ presents the vacuum permittivity coefficient (F/m), r illustrates the particle radius (m), $\zeta$ denotes the zeta potential value (V), and h presents the particles' distance in meters. KB indicates Boltzmann's constant (J/K), and T illustrates the average temperature in Kelvin.

Within the over condition, the parameter 1/k is the Debye length consistent in meters, demonstrating the EDL's thickness around the particle. This parameter is calculated through the taking-after condition.

4 $$  k = {\left( {\frac{{2{N_A}{e^2}I}}{{\varepsilon {\varepsilon_0}{K_B}T}}} \right)^{0.5}} $$

Here, N_A_ is Avogadro's number, e denotes the electron's electric charge, and I refers to the electrolyte's ionic strength (mole/Liter), calculated through the equation below.

5 $$  I = \frac{1}{2}\sum\limits_{i = 1}^n {{C_i}Z_i^2}  $$

C represents the target ion concentration in moles per liter (M), while Z denotes the electric charge of the ion. By utilizing the equations above, the energy levels of various particles can be computed relative to their distance from one another. These energy level values are then graphed against particle separation in various complexes such as kaolin-kaolin or kaolin-quartz. By utilizing the generated illustration alongside the given equation, it becomes feasible to forecast the overall interplay force present between differing particles.

6 $$  F = - \frac{dU}{{dh}}  $$

Thus, the force direction can be identified according to the slope of the energy diagram. The presence of a significant hump in the diagram indicates that the colloid is stable and the particles are not agglomerated. In contrast, a uniform diagram illustrates particle agglomeration.

### Experimental section

#### Water samples

Various salts such as sodium chloride (NaCl), calcium chloride (CaCl_2_), magnesium chloride (MgCl_2_), and sodium sulfate (Na_2_SO_4_) were used to make formation water (FW) and low salinity water samples. Table 1 illustrates the composition of different waters and their characteristics.

**Table 1:** The composition of brines and their physical characteristics.

	Na (mM)	Ca (mM)	Mg (mM)	Cl (mM)	SO_4_ (mM)	IS (mM)	TDS (ppm)	pH	viscosity at 60C
FW	3559.6	180.2	209.9	4198.9	70.4	4800	250,000	4.9	0.45
Na10SW	10.0			10.0		10	585	5.9	0.41
Ca10SW	0.0	10.0		20.0		30	1110	5.8	0.42
Na50SW	50.0			50.0		50	2925	5.6	0.43
Ca50SW		50.0		100.0		150	5550	5.6	0.43

All brines were made in the laboratory. For this purpose, the required amount of salt was slowly added to the distilled water and mixed with a magnetic stirrer for 24 h to prevent the formation of sediments. Finally, the brine fluid was filtered by filter paper.

#### Oil samples

Here, two samples of crude oil gathered from the southwestern oil fields of Iran were tested. The characteristics of tested samples are presented in Table 2.

**Table 2:** Characteristics of used oil samples.

Specification	Unit	Oil(A)	Oil(B)
Density	API	21	29
Viscosity @ 60 °C	cP	10.1	6.5
Saturates	wt%	40.1	51.3
Aromatics	wt%	38.7	23.4
Resins	wt%	7.8	7.1
Asphaltene	wt%	13.3	1.8
Total acid number (TAN)	mg-KOH/g-oil	1.52	0.03

Based on the table above, oil(A) is richer in aromatic and asphaltic molecules than oil(B). Timothy et al. illustrated that the concentration of aromatic and asphaltic functional groups highly depends on oxygenated and hydrogenated groups in oil composition. Hence, oil(A) contains more acidic functional groups than oil(B). A total acid number (TAN) experiment was conducted to bring concrete evidence, which confirmed the fact.

#### Zeta potential measurement

This work utilized a Zetasizer Nano ZS (Malvern Instruments, UK) to identify the potential in the zeta layer of various particles in different brine samples. This setup utilizes electrophoretic light scattering to identify the value.

This test measures the amount of electric potential on the surface of suspended particles. Therefore, the particles should be well suspended in the aqueous solution. Therefore, 6000 rpm rod mixers were used for 20 min to prepare the measured samples. After the mixing process for clays, sands, and oil samples, the uniformity of the fluid was visible, and this fluid was used to measure the zeta potential.

#### Interfacial tension (IFT) measurement

This work utilized the Pendent drop approach to determine the interfacial tension (IFT) between various fluids. The setup in this regard consists of various devices, such as an online image-capturing camera, a glass for the lighter liquid, and a narrow syringe pump. The IFT between oil droplets and different brine samples was determined in this study. In this experiment, an oil droplet was dropped into the brine, the bulk fluid. The injection was performed smoothly and slowly. After reaching the equilibrium, the injected droplet's image was captured and analyzed. In this study, IFT experiments have been conducted at 60 °C.

#### Wettability measurement

The impact of different brines on the rock slices' wettability was determined through a sessile drop experiment. Rock slices were prepared via a Hitti DD130 rock cutter, and the roughness of their facing was smoothed through ultra-fine sandpaper. Afterwards, the smoothed slices were washed in soxhelet via methanol to eliminate salts and then dried at 100 °C. The smooth and polished slices were immersed in crude oil for two weeks at 60 °C to approach an oil-wet condition. Aged flat pellets were then immersed in various brines to investigate their effect on wettability alteration.

Then, slices were placed on top of a chamber filled with brine. An oil droplet was poured on the facing of the slices, and a photograph was taken after stabilizing the droplet on the slice. The wettability state of the slices was determined Based on the droplet's shape. It should be noted that the contact angle measured through this experiment refers to the apparent contact angle, and to minimize the effect of hysteresis, the experiment should be conducted very slowly^46^.

#### Sandpack flooding experiment

The Pars Ore company provided quartz grains and kaolinite powder. A disk mill mortared quartz grains. The compounds of quarts and kaolinite were determined through an X-ray diffraction test after removing impurities, as illustrated in Table 3.

**Table 3:** Properties of kaolinite used to prepare sandpacks.

Specification	Mass%
Kaolinite	99
Chloride	0.025
Sulfate	0.1
Heavy metals	0.005
Average particle size 3 µm	

**Fig. 1:**
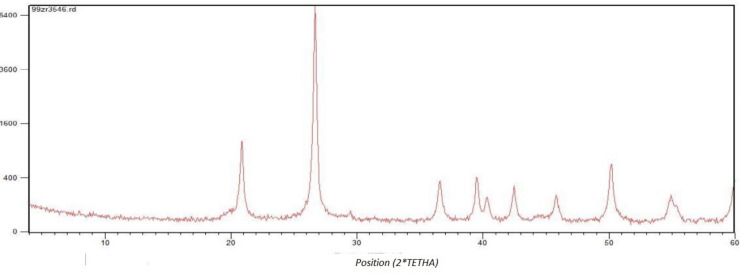
XRD analysis graph of sand particles.

XRD analysis was also conducted to characterize sand particles used in this study. Figure 1 shows the XRD results obtained from sand particle analysis, showing that 97% of the rock sample is quartz and 1.5% is clay minerals.

Based on a two-step procedure, the impurities were eliminated through rock samples. Firstly, rock powder was washed with toluene in soxhelet for 4 h to eliminate all organic impurities. Acetone was then utilized to wash toluene and soluble organic materials. Deionized water was then implemented to eliminate acetone and salts. At the end of this step, the washed powders were dried at 80 °C for 1 week.

The next step, conducted only on a quartz sample, removed inorganic materials through 15% HCl acid. Afterward, the quartz grains were washed through distilled water to eliminate the acid and achieve the inlet water's pH at the outlet.

##### Sandpack preparation procedure

Sandpacks were used instead of plugs taken from shaly sand cores. To do this, kaolinite and quartz grains were sieved to obtain the desired sizes. The mean size of kaolinite grains was 3 µm. Thus, grains were sieved through a 400 mesh to make sure that the grains were not agglomerate. Quartz grains were used in various sizes, including 177 to 250 µm, 125 to 177 µm, and 74 to 125 µm.

The sieved grains were packed in a rubber sleeve with a diameter of 1 cm. A 400 No. mesh screed was glued to the sleeve outlet to hinder sandpack disassociation. The grains that were sieved were packed in three steps. The biggest grains were poured into the sleeve in the first step, and in the last step, the smallest grains were packed.

The noteworthy point is that the inner diameter of the sandpack was 1 inch, which is considered in all calculations. However, the outer diameter is 1.5 inches, which fits in a regular core plug holder.

##### Sandpack flooding setup

**Fig. 2:**
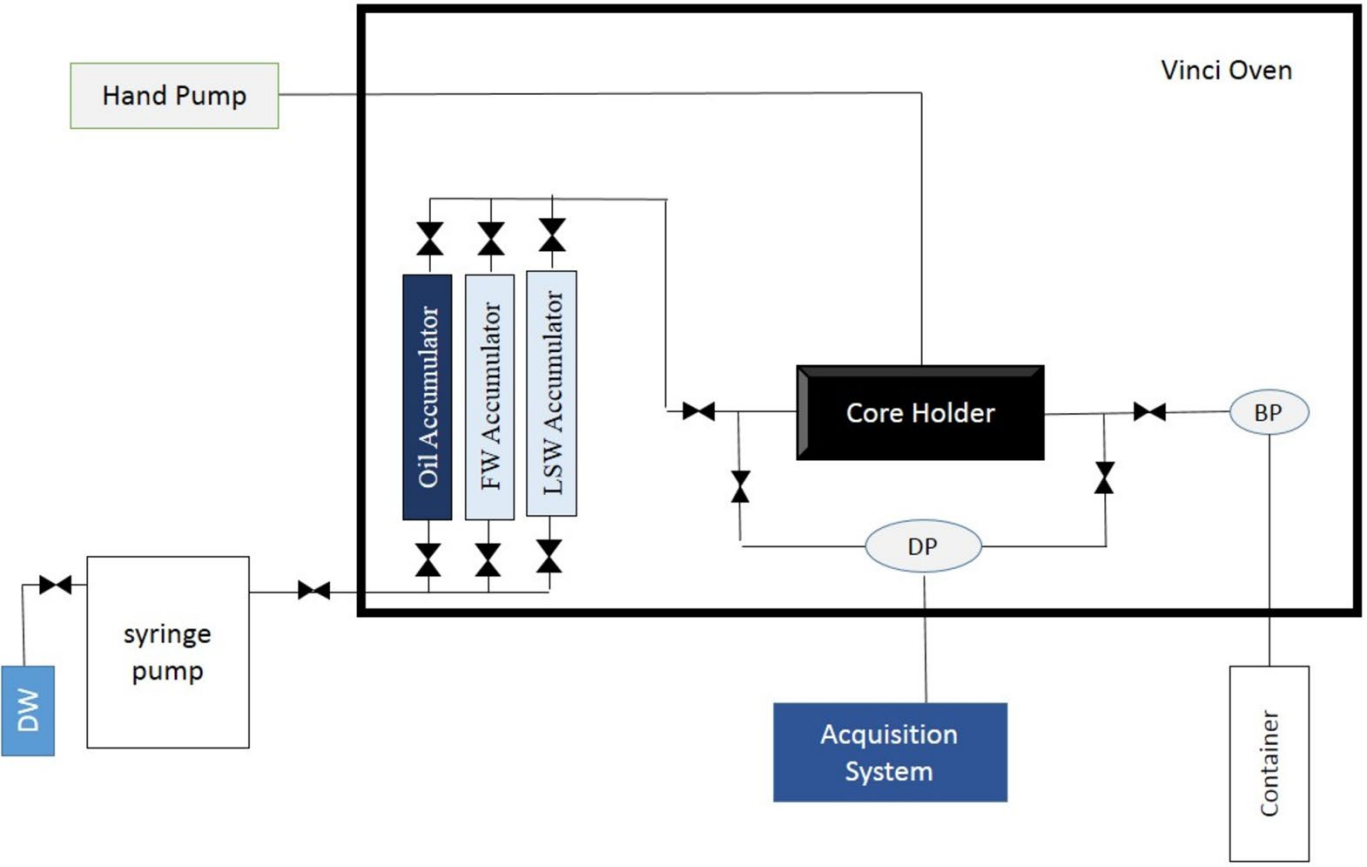
Schematic of core flooding apparatus^47^.

A flooding test was conducted to assess the efficiency of low salinity water samples in raising the recovery factor in shaly sandstones. The apparatus used for this step is illustrated in Fig. 2. Various devices were used to assemble the apparatus, including a syringe pump, three cylinders for various fluids, a core plug holder, and two pressure indicator transmitters to measure the differential pressure (dP).

Along with the test, the low salinity water samples were flooded into the shaly sandpacks, and the dp across the sandpacks was recorded. The syringe pump controlled the injection rate, and the pressure transmitters were used to measure the pressure drop across the sandpacks as the low salinity water samples flowed through them. The vacuum pump was used to maintain a constant and zero outlet pressure. The experiment was performed for various low salinity water samples, and the results were analyzed to evaluate their ability to improve RF in shaly sandpacks. In this study, all flooding experiments have been conducted at 60 °C.

Firstly, the dried and cleaned sandpacks were placed into the core holder. In the next step, they were saturated with FW. The porosity of the sandpacks can be determined by the obtained data (see Eq. 7).

7 $$  \varphi = \frac{{{V_i} - {V_o}}}{{V_b}}  $$

V refers to the fluid volume in which the index of i and o pertain to the inlet and outlet, and the index b is bulk.

To measure the absolute permeability of sandpacks, they were saturated and then flooded by formation water. The magnitude of this parameter was obtained from Eq. (8) (Darcy equation).

8 $$  K = \frac{q\mu l}{{A\Delta p}} $$

In the core flooding experiment, the injection flow rate (q) was calculated based on the viscosity of the flooded brine (µ), the length (l) and area (A) of the core plug, and the pressure difference (∆P) between the inlet and outlet of the plug.

After determining the injection flow rate, the sandpack was saturated with brine until it reached irreducible water saturation (S_wir_). Brine-saturated sandpacks were flooded by crude oil samples to achieve this condition.

The produced oil volume was plotted versus the time to investigate the impact of different parameters on crude oil recovery. The RF for different scenarios was determined through Eq. (9).

9 $$  RF = \frac{{{V_{produced}}}}{{{V_{IOIP}}}}  $$

Fine migration causes the permeability reduction in the porous medium, and as a result, the injection pressure increases while fluctuating. Therefore, it is necessary to conduct a statistical study to compare the intensity of fine migration in different sandpacks. This work calculates and determines the moving average for pressure data over time. Then, the deviation of each data is calculated concerning this moving average, and the result shows the mean absolute deviation (MAD), which can be used to compare the intensity of fine migration.

10 $$  MAD = \;\sum {\left| {\frac{{{p_t} - MA{P_t}}}{n}} \right|}  $$

In Eq. (10), P_t_ is the magnitude of pressure at a specific time, and MAP_t_ denotes the magnitude of calculated moving average pressure.

## Results and discussion

Here, the zeta potential measurement results and particle size determination tests are discussed. The force between particles for various conditions is modeled according to the DLVO theory. The impact of different parameters, including interparticle force, ion type, and concentration on the oil/brine IFT and wettability alteration, is identified.

In the end, fluids are synthesized according to the results of the batch experiments to maximize the oil recovery factor. This process is surveyed, and the oil recovery is recorded. The obtained data is investigated to determine the effect of the injected fluids in oil recovery augmentation.

### Interparticle interaction

**Fig. 3:**
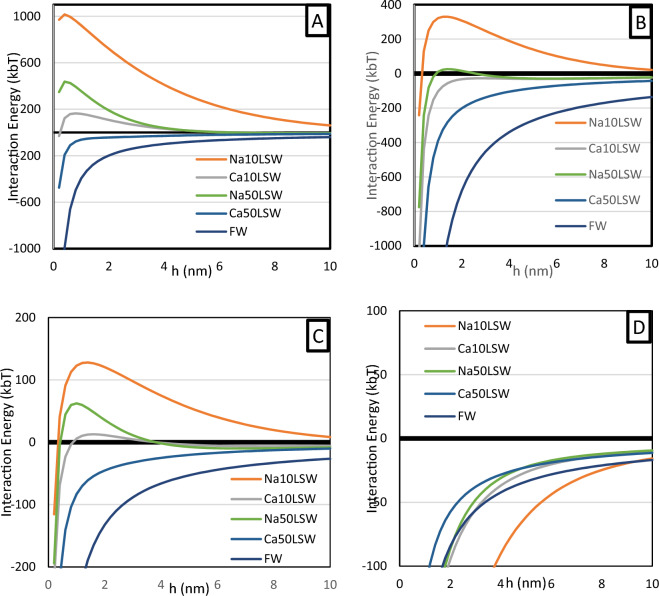
Interparticle interplay illustration in the complex (**A**) kaolinite-kaolinite (**B**) kaolinite-quartz (**C**) Oil(A)-quartz (**D**) Oil(B)-quartz.

According to the procedures above, measuring zeta potential and particle size calculates the interaction energy between particles in various systems. Figure 3 plots the magnitude of energy versus interparticle distance.

Based on Fig. 3A, kaolinite particles in FW attract each other, which leads to sedimentation. However, using low salinity water stimulates repulsion force between particles. This figure also illustrates that the repulsive force between kaolinite particles becomes dominant with a further reduction of water ionic strength by eliminating divalent cations. Thus, kaolinite particles in Na50LSW, Na10LSW, and Ca10LSW water samples are expected to repel each other, leading to a homogenous dispersion.

Figure 3B illustrates that the presence of divalent cations leads to a dominant attraction force between quartz and kaolinite particles. Therefore, quartz grains attract kaolinite particles in the FW, Ca50LSW, and Ca10LSW bulk phases. However, the interparticle forces of this complex in Na10LSW and Na50LSW are repulsive.

Figure 3C illustrates that Oil(A), which contains large amounts of acidic functional groups, behaves like kaolinite during dispersion in water. The oil droplets only attract quartz grains in the presence of formation water or water samples with divalent cations higher than 50 mM. Thus, when the concentration of these cations is low, the interparticle force in the oil-quartz complex is repulsive.

In contrast, Fig. 3D illustrates that the interparticle force between oil and quartz highly depends on the oil composition and its TAN. Unlike Oil(A), Oil(B) tends to be adsorbed on the facing of quartz particles under any conditions.

In general, it can be seen in this figure that using ten mM of monovalent ions has no substantial effect on the interparticle forces diagram, and the hump is apparent under any condition. However, by increasing the ionic strength of the fluid to the extent that it contains 50 mM of divalent cations, the hump is eliminated under any condition and makes them uniform. Therefore, investigating this concentration range is critical, and researchers must give it importance.

### The results of the IFT measurement

This research measured the effect of injection water composition on the IFT of Oil(A) and Oil(B) samples. The results are presented in Table 4.

**Table 4:** IFT values obtained for different brine/oil complex.

Oil sample	Water sample	IFT (mN/m)
Oil(A)	FW	41.4
Oil(A)	Ca50LSW	40.2
Oil(A)	Ca10LSW	38.2
Oil(A)	Na50LSW	37.4
Oil(A)	Na10LSW	36.3
Oil(B)	FW	33.4
Oil(B)	Ca50LSW	33.2
Oil(B)	Ca10LSW	32.9
Oil(B)	Na50LSW	33
Oil(B)	Na10LSW	32.6

The table illustrates that injecting Ca50LSW and Na50LSW into the oil samples significantly reduces IFT compared to FW. The IFT reduction is more significant for Oil(A) than Oil(B). Injecting Ca10LSW and Na10LSW into the oil samples also reduces IFT, but the reduction is less significant compared to Ca50LSW and Na50LSW.

The results suggest that low salinity water injection can effectively increment RF by reducing the IFT between oil and brine. The effectiveness of this mechanism may depend on the oil composition and its TAN. Oil samples with a higher TAN, such as Oil(A), may experience a more significant reduction in IFT than oil samples with a lower TAN, such as Oil(B).

The coefficient of variation (CV) was calculated for both oil(A) and oil(B) IFT data. CV for oil(A) data was 5.36, however, it was 0.92. The big difference in this parameter shows that the IFT of the first oil sample varies in different water compositions, but the IFT for the second sample is not intensely sensitive to the water composition. The data in Table 4 illustrate that the reduction in water salinity reduces the concentration of divalent cations, making a slight decline in the IFT value. This reduction is more noticeable when oil(A) is used. So, using Na10LSW reduces the IFT value from 41.4 to 36.3 mN/m. The effective mechanism of this IFT reduction is EDL expansion. The total acid number (TAN) in the Oil(A) sample is high due to acidic functional groups. Thus, the oil droplets' EDL expands with the reduction in the bulk water salinity. EDL Expansion results in a repulsive force between oil droplets, which makes them smaller and thus lowers the oil–water IFT^48,49^. Also, oil's acidic functional groups are slightly soluble in water^50^. Decreasing the salinity of water and the components dissolved in it increases their solubility, resulting in IFT reductions. However, the reduction of IFT in oil (B) is meager. This is due to the absence of acidic functional groups in this oil sample, and the brine/oil IFT value in this complex is not dependent on water salinity. It should be noted that IFT reduction is not a primary mechanism in low salinity water flooding, and wettability alteration and fine migration phenomenon are much more effective than IFT reduction.

### Wettability alteration results

**Fig. 4:**
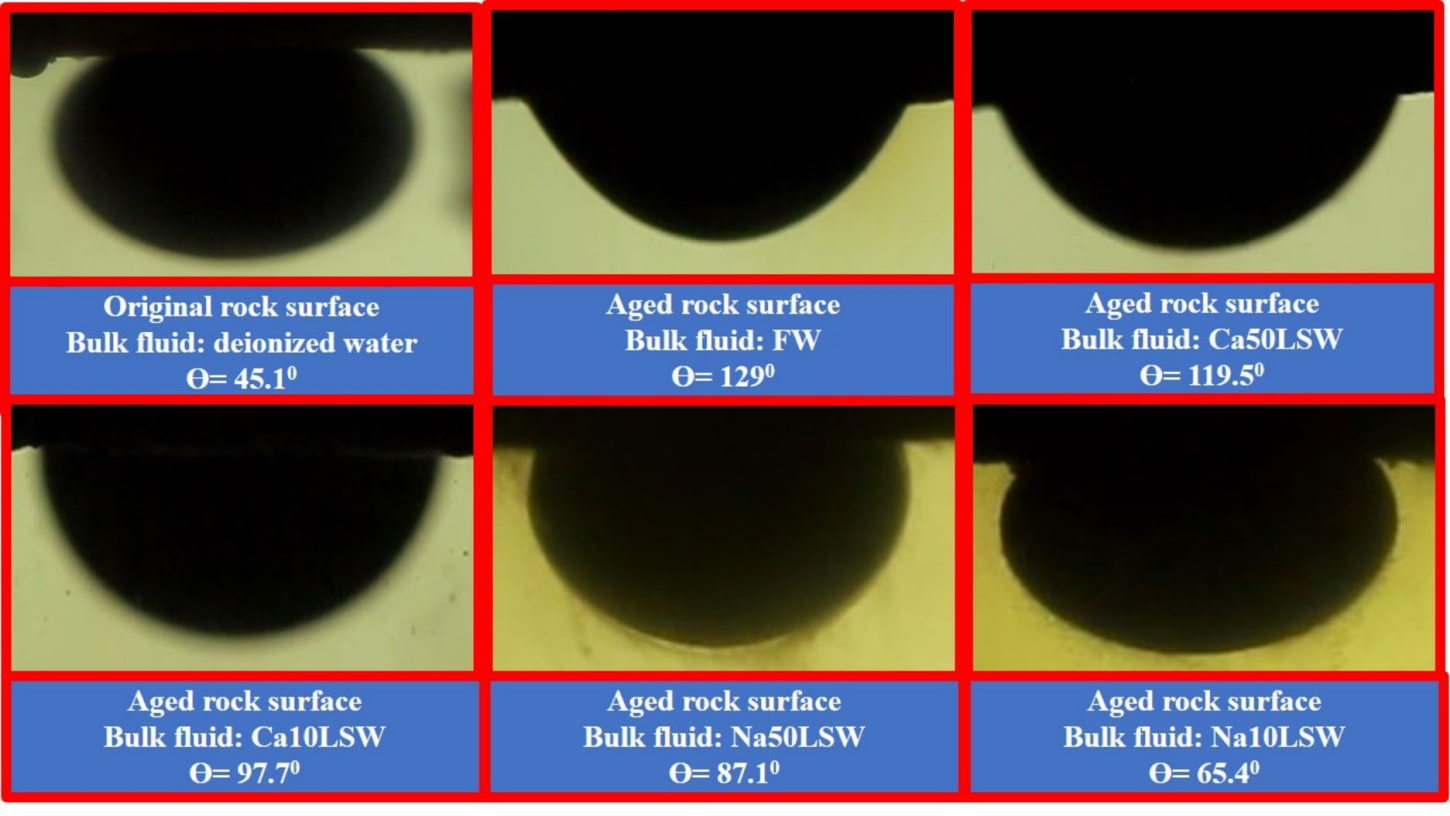
Contact angel images of an oil drop on a quartz slice in different conditions.

**Fig. 5:**
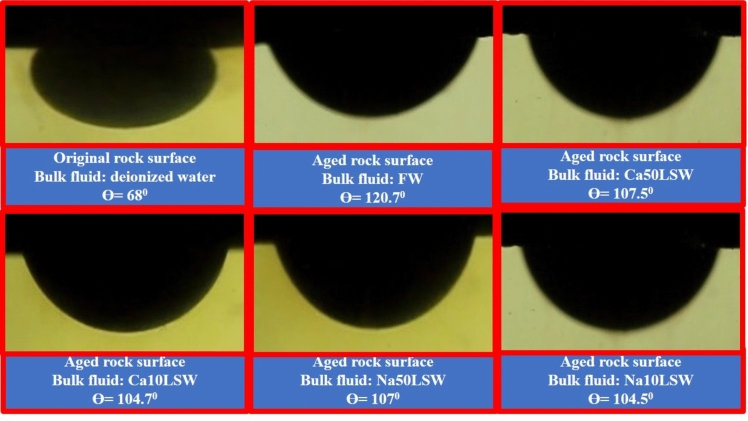
Contact angel images for brine/oil complex (B) in different bulk fluids.

The contact angle test was performed to evaluate the effect of different brines on the wettability of reservoir rock. The figures obtained for different oil samples (oil(A) and oil(B)) are illustrated in Figs. 4, 5, respectively.

Figure 5 illustrates that the quartz facing is initially water-wet, with an oil apparent contact angle^46^ of 45.1°. However, after aging the quartz slices for 14 days, the facing becomes oil-wet, with an apparent contact angle of 129°. This wettability alteration is attributed to acidic functional groups in the oil composition, which adsorb onto the silicate facings and change the facing's wettability to oil-wet.

When aged rock is exposed to low-salinity water, wettability alteration can occur for two reasons. Firstly, EDL expansion in quartz slices and oil droplets can lead repulsive forces to prevail in the quartz-oil(A) complex, separating oil droplets from the rock facing. This repulsive force results from reduced water salinity, significantly reducing the concentration of divalent cations in the water. Secondly, the solubility of functional groups in water increments as the water salinity reduces. This incremented solubility leads the functional groups to dissolve in the water, separating oil droplets from the rock facing.

**Fig. 6:**
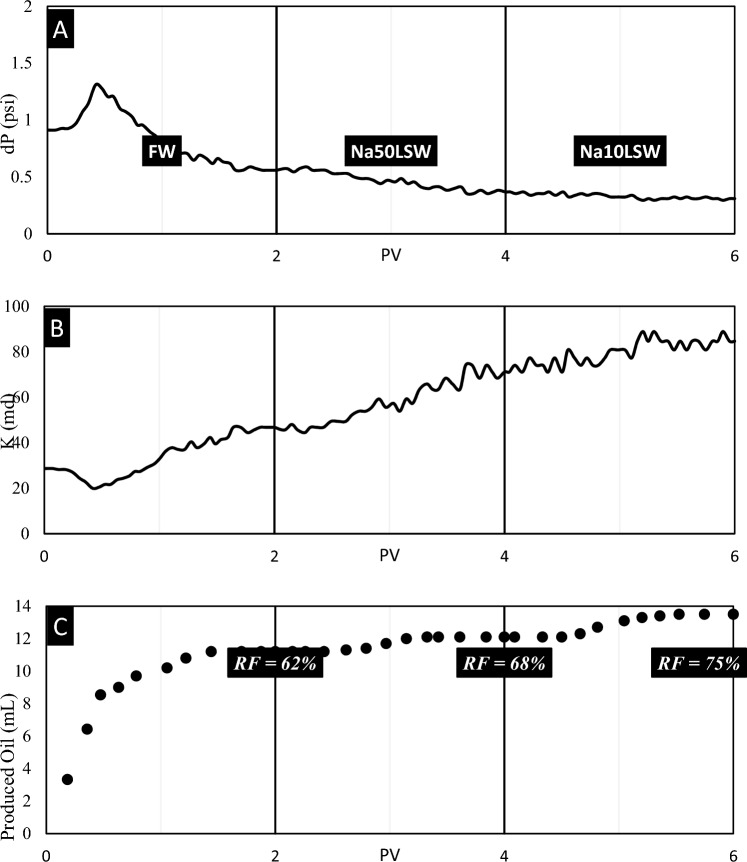
The results of (**A**) pressure, (**B**) permeability, and (**C**) recovery volume obtained from flooding of sandpack No. 1 by FW, Na50LSW, and Na10LSW.

Figure 6 illustrates that the wettability alteration of the complex containing Oil(B) is minimally affected by the reduction of water salinity. This may be due to differences in the composition of Oil(B) compared to Oil(A), leading to different adsorption and interplay behavior with the rock facing.

Overall, wettability alteration is essential for improving oil recovery in low salinity water injection. The mechanism is complex and can be affected by various factors, including the oil composition, rock properties, and injection water composition. Thus, careful evaluation and optimization are necessary to maximize the potential benefits of this technique.

Figure 5 displays contact angle images for the brine/oil complex (B) in different bulk fluids. Similar to the previous figure, aging the rock with Oil(B) increases the oil wetness of the rock face, but the contact angle increment is less significant compared to Oil(A). This observation can be attributed to the lower concentration of acidic functional groups in Oil(B) compared to Oil(A).

Moreover, reducing water salinity and removing divalent cations had a limited effect on the wettability alteration of the complex containing Oil(B). This could be due to the absence of repulsive forces between the oil and the rock facing resulting from EDL expansion or the low concentration of functional groups in Oil(B), which cannot alter the wettability by dissolving in water.

In summary, the wettability alteration mechanism in low salinity water injection can be influenced by various factors, such as the oil composition, rock properties, and injection water composition. Thus, it is crucial to thoroughly evaluate and optimize these factors to achieve optimal results with low salinity water injection in improving oil recovery.

### Sandpack flooding results

Eight quartz sandpacks containing zero and 5 wt% of kaolinite particles were prepared. The sandpacks' permeability and porosity were measured by injecting formation water (FW) at a rate of 0.1 ml/min (equivalent to 3 ft/day). Based on the established procedure, the sandpacks were saturated with Swir and prepared for flooding, as outlined in Table 5.

**Table 5:** Characteristics of tested sandpacks.

No	Clay Con (%)	Length (cm)	Diameter (In)	Porosity (%)	Permeability (md)	S_oi_	Oil sample
1	0.0	12	1	29.6	163.6	87.0	A
2	0.0	12	1	29.9	171.2	82.0	B
3	0.0	12	1	29.9	165.4	86.6	A
4	0.0	12	1	29.8	166.1	81.6	B
5	5.0	12	1	27.7	61.6	83.7	A
6	5.0	12	1	28.1	61.8	84.8	B
7	5.1	12	1	29.0	66.2	84.0	A
8	5.1	12	1	28.4	64.6	84.4	B

The CV parameter for no clay samples is 0.47%, and it is 1.9% for clay-rich sandpacks, which shows that the process is repeatable and there is no significant variation in the porosity in identical samples. In the next step, to check the effect of different factors on the recovery factor and the fine migration phenomenon, each of these sandpacks was flooded by various scenarios.

Test No. 1: Successfully, Sandpack No. 1 was flooded by FW, Na50LSW, and Na10LSW samples. The results of this test are illustrated in Fig. 6.

**Fig. 7:**
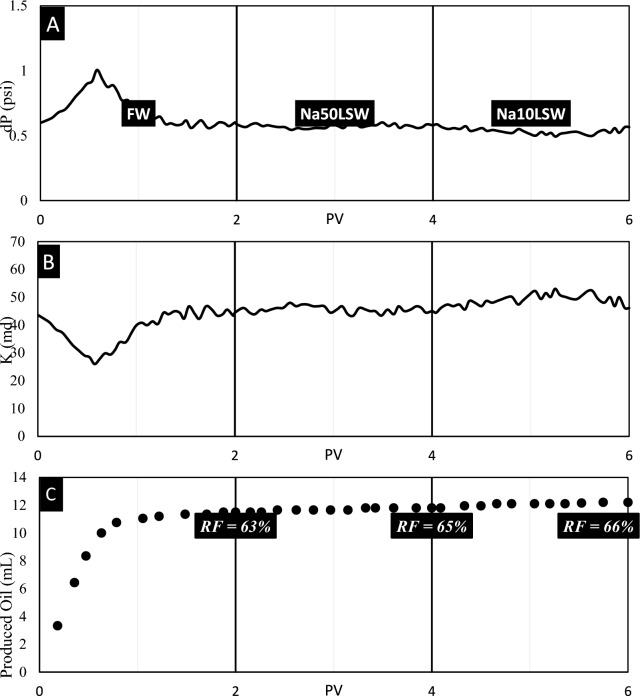
The results of (**A**) pressure, (**B**) permeability, and (**C**) recovery volume obtained from flooding of sandpack No. 2 by FW, Na50LSW, and Na10LSW.

During the initial stages of sandpack flooding, the injection pressure increments until it reaches a hump, after which it reduces. This hump is resulted from the water phase being trapped behind the oil bank, and the pressure starts to reduce when the water breaks through. The pressure becomes stable after injecting about 1.6 PV, and no significant changes occur in the sandpack's parameters. However, before this region, the pressure drop and effective permeability increment due to oil exiting from the sandpack outlet, leading to an increment in water saturation in the porous media, as illustrated in Fig. 7C.

When Na50LSW is injected into the sandpack, the injection pressure starts to reduce, and the permeability increments after about 0.5 PV. This is likely due to the separation of some oil from the rock due to Na50LSW entering the sandpack, increasing water saturation and effective permeability of the water phase. The positive effect of Na50LSW on oil recovery is observed in IFT and wettability alteration tests, and the recovery factor increments by 6% at the end of the injection.

Injecting Na10LSW leads to a remarkable increment in the recovery factor and permeability. This is because Na10LSW entering the porous media makes the facing of the oil droplets and rock charge, resulting in a prevailing repulsive force. IFT and wettability data also support this finding.

In summary, the injection of Na50LSW and Na10LSW can positively impact oil recovery through their effects on water saturation, effective permeability, and interfacial tension. However, the optimal injection strategy may depend on various factors, such as reservoir characteristics, oil properties, and injection water composition, and careful evaluation and optimization are necessary to achieve the best results.

According to Fig. 6, pressure decreases smoothly over time, which shows that no fine migration occurs in this porous media. To make a better index for comparison with other experiments, the parameter MAD for pressure data obtained from this experiment was calculated, equaling 0.0127.

Test No. 2: Sandpack No. 2 was displaced by Oil(B) to S_wir_. Then it was flooded by FW, Na50LSW, and Na10LSW, successively. The results of this test are illustrated in Fig. 7.

Contrary to the previous experiment, the recovery factor did not increase when these low salinity water samples were injected. In the wettability alteration and IFT test, it was also observed that these water samples are unsuitable for improving this oil's recovery factor. The parameter MAD for pressure data was equal to 0.0119, which is very low and shows a negligible fluctuation in data, illustrating no fine migration status.

**Fig. 8:**
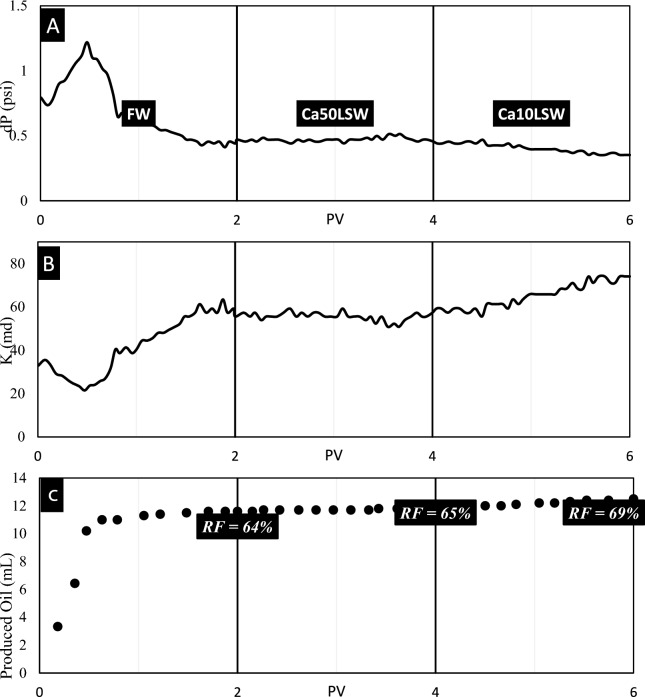
The results of (**A**) pressure, (**B**) permeability, and (**C**) recovery volume obtained from flooding of sandpack No. 3 by FW, Ca50LSW, and Ca10LSW.

Test No. 3: In this test, the third sandpack was flooded by FW, Ca50LSW, and Ca10LSW. Figure 8 illustrates the results of this test.

Figure 8 clearly illustrates that the injection of Ca50LSW does not improve conditions. The previous experiments also discussed that this concentration of divalent cations resulted in a severe contraction of EDL in matrix particles and oil droplets. As a result, wettability and IFT do not improve. However, by reducing the concentration of this cation to 10 mM, EDL expansion occurs, and RF increments as repulsive forces dominate. Here, the permeability value incremented from 36 to 45 md, which can be affected by various factors such as wettability alteration, IFT reduction, and Sw increment. The MAD parameter for pressure data obtained from this experiment was 0.0108, which shows a smooth pressure change over time.

**Fig. 9:**
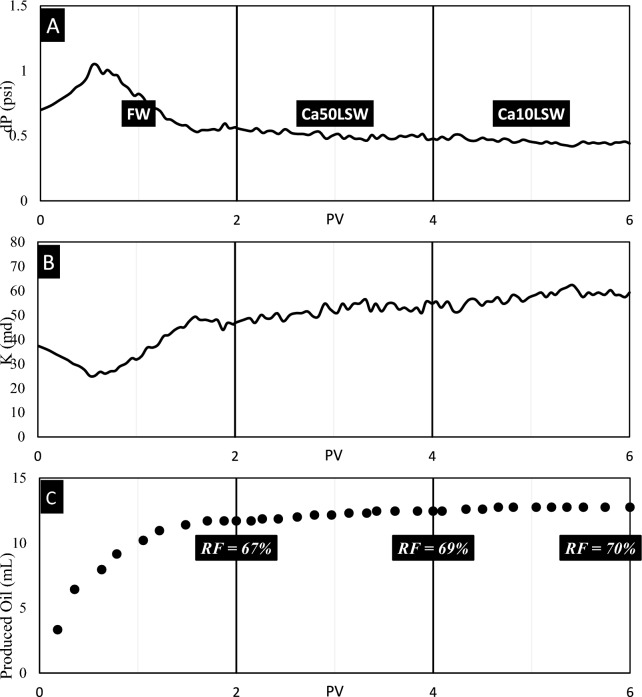
The results of (**A**) pressure, (**B**) permeability, and (**C**) recovery volume obtained from flooding of sandpack No. 4 by FW, Ca50LSW, and Ca10LSW.

Test No. 4: Sandpack No. 4 was flooded by FW, Ca50LSW, and Ca10LSW successively. The data obtained from this experiment is illustrated in Fig. 9.

The data in this figure illustrate that reducing water salinity is ineffective in raising the RF of Oil(B). Also, the wettability and IFT data illustrated that the expansion of EDL does not increment the values of repulsive forces by reducing the salinity of injected water. The MAD parameter is 0.0111 showing smooth change in pressure; therefore, it is a convincing reason that fine migration does not occur.

**Fig. 10:**
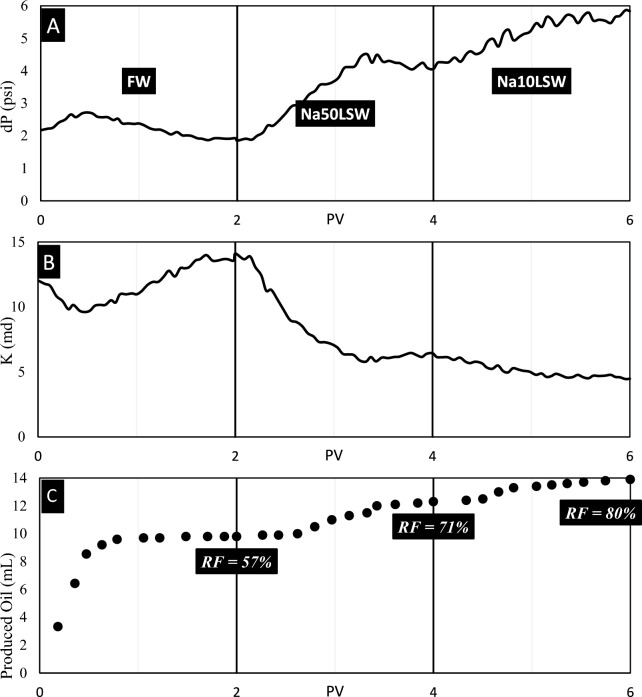
The results of (**A**) pressure, (**B**) permeability, and (**C**) recovery volume obtained from flooding of sandpack No. 5 by FW, Na50LSW, and Na10LSW.

Test No. 5: In this test, sandpack No. 5, which contains five wt% kaolinite, was flooded by FW, Na50LSW, and Na10LSW. The results of this test are illustrated in Fig. 10.

Based on the data in this figure, it is clear that the amount of RF increments with the injection of Na50LSW. This paper previously explained that Na50LSW can alter the matrix's wettability to water-wet. Thus, the positive effect of this brine in increasing RF is apparent. However, there is a fundamental difference here with the increment of RF, which increments the S_w_ in the sandpack, and the value of K_eff_ for the water phase reductions. Also, the increment in RF for this experiment (from 57 to 80%) is much higher than in experiment number 1 (from 62 to 75%), where kaolinite particles were absent in the sandpack. Thus, injecting this water and Na10LSW into the sandpack is a more effective phenomenon than wettability alteration. Based on the analysis of interparticle forces in the kaolinite-quartz complex, it was found that these brines result in repulsive forces prevailing. Thus, this phenomenon is fine migration, which increments the recovery factor.

Statistical analysis of pressure data in this experiment shows a moving average of pressure increases over time. Furthermore, the magnitude of MAD for pressure data was equal to 0.0636, which is much higher than the previous experiment and shows fluctuation in data. As previously discussed, pressure increments with fluctuation are a clue to the occurrence of fine migration.

Test No. 6. Here, sandpack No. 6 containing 5 wt% kaolinite particles and Oil(B) is flooded by FW, Na50LSW, and Na10LSW.

**Fig. 11:**
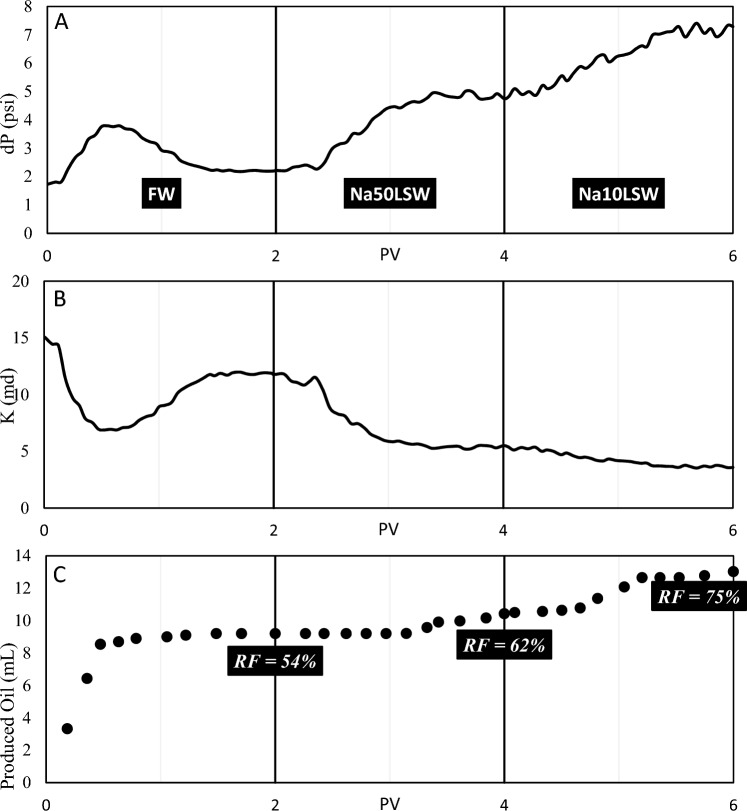
The results of (**A**) pressure, (**B**) permeability, and (**C**) recovery volume obtained from flooding of sandpack No. 6 by FW, Na50LSW, and Na10LSW.

**Fig. 12:**
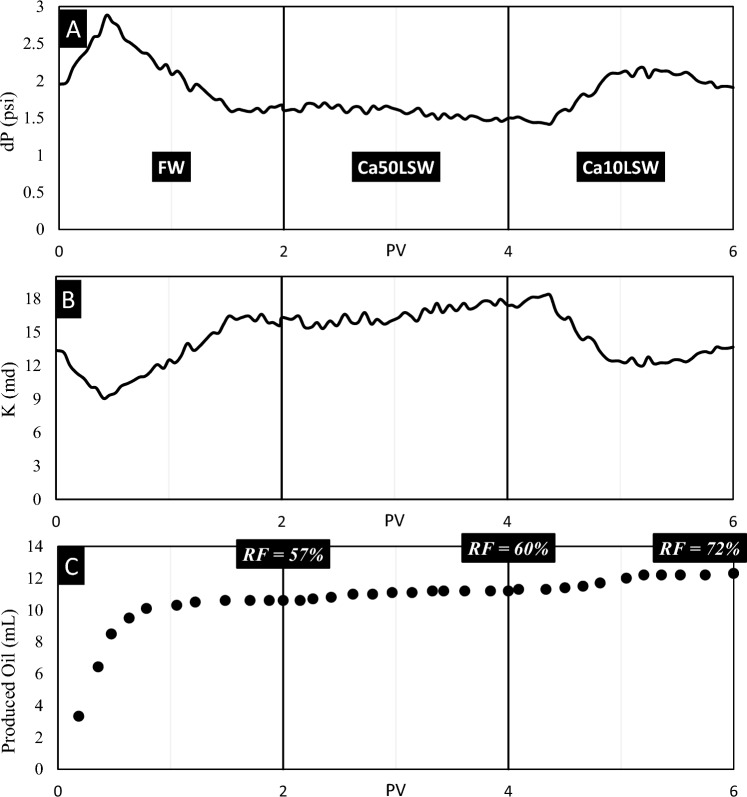
The results of (**A**) pressure, (**B**) permeability, and (**C**) recovery volume obtained from flooding of sandpack No. 7 by FW, Ca50LSW, and Ca10LSW.

Figure 11 illustrates that injection of Na50LSW and Na10LSW increments RF. Also, in this process, contrary to experiments 1–4, the increment in RF is accompanied by a reduction in permeability. Also, it was found earlier in this article that these brines cannot alter the wettability of oil (B) and quartz. Also, IFT changes can be ignored for this oil sample. Thus, it is clear that fine migration leads this oil to be swept from the pores. Figure 12 illustrates that this phenomenon increments RF by 14%. While the only possible phenomenon in this test is fine migration, it has had an acceptable performance and has resulted in RF improvement even more than wettability alteration and IFT reduction. The parameter MAD was equal to 0.0692, which shows fluctuation in pressure data.

Test No. 7: In this test, sandpack No. 7, which contains 5 wt% kaolinite and oil(A), was flooded by FW, Ca50LSW, and Ca10LSW. The results are illustrated in Fig. 12.

It can be seen here that when the concentration of divalent cations in the injection water is high, the benefits of low salinity water injection do not occur. Neither fine migration nor wettability alteration nor IFT reduction has happened. For this reason, the amount of RF did not improve much with the injection of Ca50LSW. However, with the reduction in the concentration of divalent cations, the number of RF increments. Considering that the rise in oil production from sandpack coincides with the reduction in permeability, it is sure that fine migration is also effective. The parameter MAD for pressure data was 0.0302 in this experiment, which shows that a lower degree of fine migration occurred.

**Fig. 13:**
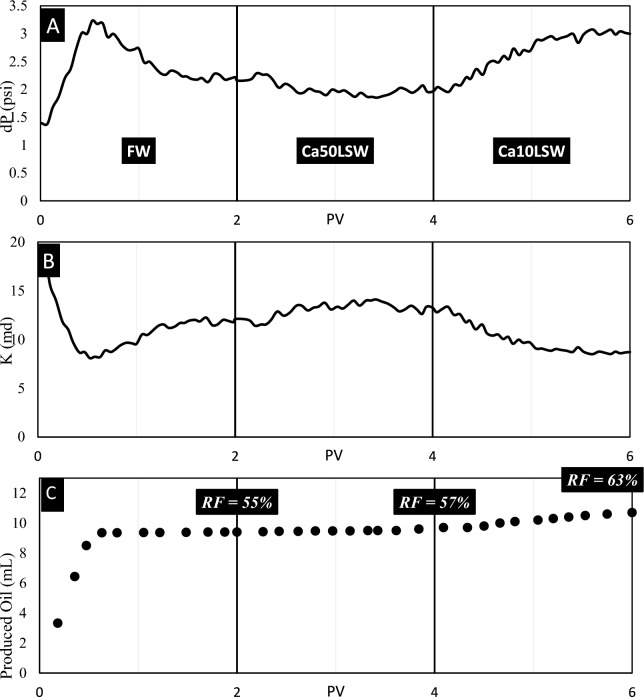
The results of (**A**) pressure, (**B**) permeability, and (**C**) recovery volume obtained from flooding of sandpack No. 8 by FW, Ca50LSW, and Ca10LSW.

Test No. 8. Sandpack No. 8 was flooded by FW, Ca50LSW, and Ca10LSW; the results are illustrated in Fig. 13.

Figure 13 illustrates that RF starts to increase when the concentration of divalent cations reduces to 10 mmol. Meanwhile, none of the wettability alteration and IFT reduction mechanisms are expected for this condition. Also, because the increment in RF occurs after the reduction in permeability, it is evident that fine migration is the effective mechanism. The parameter MAD for pressure data was 0.0412, which shows partial fine migration.

## Conclusion

This research first studied the impact of various parameters such as salinity, divalent cation concentration, and oil type on interparticle forces in a clay-rich porous media. The below results were obtained from this step.

1. Lowering salinity stimulates repulsive forces between various particles. This occurs when TDS is less than 3000 ppm and divalent cation concentration is less than ten mmol.
2. The interparticle force in the oil-quartz system is mainly related to the chemistry of the crude oil. Acidic functional groups present in oil make the oil sensitive to water salinity. Thus, reducing salinity, particularly divalent cation concentration, makes the oil droplets smaller.
3. Interparticle repulsive forces between oil droplets lead to IFT reduction. Thus, water salinity can affect the IFT value of oil with a high total acid number (TAN), but not for other oils. It should be noted that IFT reduction is less effective than wettability alteration and fine migration for oil recovery.

A core flooding experiment was conducted in the next step, which showed that.

1. Reducing the TDS of the injected brine to less than 3000 ppm and the concentration of divalent cations to 10 mM leads to fine migration in the porous media, which positively impacts the recovery factor (RF) for both types of oil.
2. Fine migration increments the RF of any oil, while wettability alteration and IFT reduction depend on the oil type in the porous media and are less effective than fine migration.

In conclusion, the study provides valuable insights into the interparticle forces and their effects on low salinity water performance, highlighting the importance of careful evaluation and optimization of injection water composition to achieve optimal results in oil recovery.

### Supplementary Information

Supplementary Information.

## Data Availability

The authors declare that the data supporting of this study are available within the paper and its Supplementary Information files.

## Abbreviations

BN: Base number
DLVO: Derjaguin, Landau, Verwey, and Overbeek
EDL: Electric double layer
EOR: Enhanced oil recovery
FW: Formation water
IFT: Interfacial tension
LSW: Low salinity water
MAD: Mean absolute deviation
RF: Recovery factor
TAN: Total acid number
XRD: X-ray differaction

## References

[1] MosalmanHaghighi, O. & MohsenatabarFirozjaii, A. An experimental investigation into enhancing oil recovery using combination of new green surfactant with smart water in oil-wet carbonate reservoir. *J. Pet. Explor. Prod. Technol.* **10**, 893–901. 10.1007/s13202-019-0741-7 (2020). 10.1007/s13202-019-0741-7

[2] Honarvar, B., Rahimi, A., Safari, M., Khajehahmadi, S. & Karimi, M. Smart water effects on a crude oil-brine-carbonate rock (CBR) system: Further suggestions on mechanisms and conditions. *J. Mol. Liq.* **299**, 112173. 10.1016/j.molliq.2019.112173 (2020). 10.1016/j.molliq.2019.112173

[3] Saw, R. K., Singh, A., Maurya, N. K. & Mandal, A. A mechanistic study of low salinity water-based nanoparticle-polymer complex fluid for improved oil recovery in sandstone reservoirs. *Colloids Surf. A* **666**, 131308. 10.1016/j.colsurfa.2023.131308 (2023). 10.1016/j.colsurfa.2023.131308

[4] Tajikmansori, A., Hossein SaeediDehaghani, A. & Haghighi, M. Improving chemical composition of smart water by investigating performance of active cations for injection in carbonate Reservoirs: A mechanistic study. *J. Mol. Liq.* **348**, 118043. 10.1016/j.molliq.2021.118043 (2022). 10.1016/j.molliq.2021.118043

[5] Namaee-Ghasemi, A., Behbahani, H.S.-Z., Kord, S. & Sharifi, A. Geochemical simulation of wettability alteration and effluent ionic analysis during smart water flooding in carbonate rocks: Insights into the mechanisms and their contributions. *J. Mol. Liq.* **326**, 114854. 10.1016/j.molliq.2020.114854 (2021). 10.1016/j.molliq.2020.114854

[6] Katende, A. & Sagala, F. A critical review of low salinity water flooding: Mechanism, laboratory and field application. *J. Mol. Liq.* **278**, 627–649. 10.1016/j.molliq.2019.01.037 (2019). 10.1016/j.molliq.2019.01.037

[7] Gomez, S., Mansi, M. & Fahes, M. In *Abu Dhabi International Petroleum Exhibition & Conference* D031S088R002 (2018).

[8] Saw, R. K., Pillai, P. & Mandal, A. Synergistic effect of low saline ion tuned Sea Water with ionic liquids for enhanced oil recovery from carbonate reservoirs. *J. Mol. Liq.* **364**, 120011. 10.1016/j.molliq.2022.120011 (2022). 10.1016/j.molliq.2022.120011

[9] Farhadi, H., Fatemi, M. & Ayatollahi, S. Experimental investigation on the dominating fluid-fluid and rock-fluid interactions during low salinity water flooding in water-wet and oil-wet calcites. *J. Petrol. Sci. Eng.* **204**, 108697. 10.1016/j.petrol.2021.108697 (2021). 10.1016/j.petrol.2021.108697

[10] Ghasemi, M., Shafiei, A. & Foroozesh, J. A systematic and critical review of application of molecular dynamics simulation in low salinity water injection. *Adv. Colloids Interface. Sci.* **300**, 102594. 10.1016/j.cis.2021.102594 (2022). 10.1016/j.cis.2021.102594 34971915

[11] Austad, T., RezaeiDoust, A. & Puntervold, T. In *SPE Improved Oil Recovery Symposium* SPE-129767-MS (2010).

[12] Smith, E. R., Medina-Rodríguez, B. X. & Alvarado, V. Influence of interfacial responses of Berea Sandstone in low-salinity waterflooding environments. *Fuel* **311**, 121712. 10.1016/j.fuel.2021.121712 (2022). 10.1016/j.fuel.2021.121712

[13] Sheng, J. J. Critical review of low-salinity waterflooding. *J. Pet. Sci. Eng.* **120**, 216–224. 10.1016/j.petrol.2014.05.026 (2014). 10.1016/j.petrol.2014.05.026

[14] Kumar Saw, R. & Mandal, A. Experimental investigation on fluid/fluid and rock/fluid interactions in enhanced oil recovery by low salinity water flooding for carbonate reservoirs. *Fuel* **352**, 129156. 10.1016/j.fuel.2023.129156 (2023). 10.1016/j.fuel.2023.129156

[15] Alameri, W., Teklu, T. W., Graves, R. M., Kazemi, H. & AlSumaiti, A. M. In *SPE Asia Pacific Oil & Gas Conference and Exhibition* SPE-171529-MS (2014).

[16] Yang, Y. *et al.* Formation damage evaluation of a sandstone reservoir via pore-scale X-ray computed tomography analysis. *J. Pet. Sci. Eng.* **183**, 106356. 10.1016/j.petrol.2019.106356 (2019). 10.1016/j.petrol.2019.106356

[17] Nasr-El-Din, H. A. *et al.* Field treatment to stimulate an oil well in an offshore sandstone reservoir using a novel, low-corrosive, environmentally friendly fluid. *J. Can. Pet. Technol.* **54**, 289–297. 10.2118/168163-PA (2015). 10.2118/168163-PA

[18] Sheng, J. J. Formation damage in chemical enhanced oil recovery processes. *Asia-Pac. J. Chem. Eng.* **11**, 826–835. 10.1002/apj.2035 (2016). 10.1002/apj.2035

[19] Chavan, M., Dandekar, A., Patil, S. & Khataniar, S. Low-salinity-based enhanced oil recovery literature review and associated screening criteria. *Pet. Sci.* **16**, 1344–1360. 10.1007/s12182-019-0325-7 (2019). 10.1007/s12182-019-0325-7

[20] Miranda, R. M. & Underdown, D. R. In *SPE Production Operations Symposium* SPE-25432-MS (1993).

[21] Zhou, Z. J., Gunter, W. O. & Jonasson, R. G. In *Annual Technical Meeting* PETSOC-95-71 (1995).

[22] Khezerlooe-ye Aghdam, S. *et al.* Mechanistic assessment of Seidlitzia Rosmarinus-derived surfactant for restraining shale hydration: A comprehensive experimental investigation. *Chem. Eng. Res. Des.* **147**, 570–578. 10.1016/j.cherd.2019.05.042 (2019). 10.1016/j.cherd.2019.05.042

[23] Aghdam, S.K.-Y., Kazemi, A. & Ahmadi, M. A laboratory study of a novel bio-based nonionic surfactant to mitigate clay swelling. *Petroleum* **7**, 178–187. 10.1016/j.petlm.2020.09.002 (2021). 10.1016/j.petlm.2020.09.002

[24] Oyeneyin, M. B., Peden, J. M., Hosseini, A. & Ren, G. In *SPE European Formation Damage Conference* SPE-30112-MS (1995).

[25] Al-Sarihi, A*. et al.* In *SPE Asia Pacific Oil and Gas Conference and Exhibition* D021S008R004 (2018).

[26] Bedrikovetsky, P., Zeinijahromi, A., Badalyan, A., Ahmetgareev, V. & Khisamov, R. In *SPE Russian Petroleum Technology Conference* SPE-176721-MS (2015).

[27] Alhuraishawy, A. K., Bai, B., Wei, M., Geng, J. & Pu, J. Mineral dissolution and fine migration effect on oil recovery factor by low-salinity water flooding in low-permeability sandstone reservoir. *Fuel* **220**, 898–907. 10.1016/j.fuel.2018.02.016 (2018). 10.1016/j.fuel.2018.02.016

[28] Bigno, Y., Oyeneyin, M. B. & Peden, J. M. In *SPE Formation Damage Control Symposium* SPE-27342-MS (1994).

[29] Zhang, L. *et al.* Particle migration and blockage in geothermal reservoirs during water reinjection: Laboratory experiment and reaction kinetic model. *Energy* **206**, 118234. 10.1016/j.energy.2020.118234 (2020). 10.1016/j.energy.2020.118234

[30] Musharova, D. A., Mohamed, I. M. & Nasr-El-Din, H. A. In *SPE International Symposium and Exhibition on Formation Damage Control* SPE-150953-MS (2012).

[31] Barnaji, M. J., Pourafshary, P. & Rasaie, M. R. Visual investigation of the effects of clay minerals on enhancement of oil recovery by low salinity water flooding. *Fuel* **184**, 826–835. 10.1016/j.fuel.2016.07.076 (2016). 10.1016/j.fuel.2016.07.076

[32] Tang, G.-Q. & Morrow, N. R. Influence of brine composition and fines migration on crude oil/brine/rock interactions and oil recovery. *J. Pet. Sci. Eng.* **24**, 99–111. 10.1016/S0920-4105(99)00034-0 (1999). 10.1016/S0920-4105(99)00034-0

[33] Zeinijahromi, A. & Bedrikovetsky, P. In *SPE Russian Petroleum Technology Conference* SPE-176548-MS (2015).

[34] Zeinijahromi, A. & Bedrikovetsky, P. In *SPE International Conference and Exhibition on Formation Damage Control* D021S010R003 (2016).

[35] Wu, Q.-H., Ge, J.-J., Ding, L. & Zhang, G.-C. Unlocking the potentials of gel conformance for water shutoff in fractured reservoirs: Favorable attributes of the double network gel for enhancing oil recovery. *Pet. Sci.*10.1016/j.petsci.2022.10.018 (2022). 10.1016/j.petsci.2022.10.018

[36] Olayiwola, S. O. & Dejam, M. A comprehensive review on interaction of nanoparticles with low salinity water and surfactant for enhanced oil recovery in sandstone and carbonate reservoirs. *Fuel* **241**, 1045–1057. 10.1016/j.fuel.2018.12.122 (2019). 10.1016/j.fuel.2018.12.122

[37] Al-Attar, H. H., Mahmoud, M. Y., Zekri, A. Y., Almehaideb, R. A. & Ghannam, M. T. In *EAGE Annual Conference & Exhibition incorporating SPE Europec* SPE-164788-MS (2013).

[38] Farhadi, H., Ayatollahi, S. & Fatemi, M. The effect of brine salinity and oil components on dynamic IFT behavior of oil-brine during low salinity water flooding: Diffusion coefficient, EDL establishment time, and IFT reduction rate. *J. Pet. Sci. Eng.* **196**, 107862. 10.1016/j.petrol.2020.107862 (2021). 10.1016/j.petrol.2020.107862

[39] Bashir, A., Sharifi Haddad, A. & Rafati, R. A review of fluid displacement mechanisms in surfactant-based chemical enhanced oil recovery processes: Analyses of key influencing factors. *Pet. Sci.* **19**, 1211–1235. 10.1016/j.petsci.2021.11.021 (2022). 10.1016/j.petsci.2021.11.021

[40] Saw, R. K. & Mandal, A. A mechanistic investigation of low salinity water flooding coupled with ion tuning for enhanced oil recovery. *RSC Adv.* **10**, 42570–42583. 10.1039/D0RA08301A (2020). 35516738 10.1039/D0RA08301A PMC9057959

[41] Al-Shalabi, E. W. & Sepehrnoori, K. A comprehensive review of low salinity/engineered water injections and their applications in sandstone and carbonate rocks. *J. Pet. Sci. Eng.* **139**, 137–161. 10.1016/j.petrol.2015.11.027 (2016). 10.1016/j.petrol.2015.11.027

[42] Khisamov, V., Akhmetgareev, V. & Shilova, T. Core tests and field case studies of successful and unsuccessful low-salinity waterfloods from four oil fields. 10.3997/2214-4609.201701703 (2017).

[43] Missana, T. & Adell, A. On the applicability of DLVO theory to the prediction of clay colloids stability. *J. Colloid Interface Sci.* **230**, 150–156. 10.1006/jcis.2000.7003 (2000). 10998299 10.1006/jcis.2000.7003

[44] Adamczyk, Z. & Weroński, P. Application of the DLVO theory for particle deposition problems. *Adv. Colloid Interface Sci.* **83**, 137–226. 10.1016/S0001-8686(99)00009-3 (1999). 10.1016/S0001-8686(99)00009-3

[45] RohemPeçanha, E. & da Fonseca de Albuquerque, M. D., AntounSimão, R., de Salles Leal Filho, L. & de Mello Monte, M. B.,. Interaction forces between colloidal starch and quartz and hematite particles in mineral flotation. *Colloids Surf. A Physicochem. Eng. Asp.* **562**, 79–85. 10.1016/j.colsurfa.2018.11.026 (2019). 10.1016/j.colsurfa.2018.11.026

[46] Bormashenko, E., Balter, S., Bormashenko, Y. & Aurbach, D. Honeycomb structures obtained with breath figures self-assembly allow water/oil separation. *Colloids Surf. A Physicochem. Eng. Asp.* **415**, 394–398. 10.1016/j.colsurfa.2012.09.022 (2012). 10.1016/j.colsurfa.2012.09.022

[47] Aghdam, S.K.-Y., Kazemi, A. & Ahmadi, M. Studying the effect of various surfactants on the possibility and intensity of fine migration during low-salinity water flooding in clay-rich sandstones. *Results Eng.* **18**, 101149. 10.1016/j.rineng.2023.101149 (2023). 10.1016/j.rineng.2023.101149

[48] Aghdam, S. K.-y., Kazemi, A. & Ahmadi, M. Theoretical and experimental study of fine migration during low-salinity water flooding: Effect of brine composition on interparticle forces. In *SPE Reservoir Evaluation & Engineering* 1–16. 10.2118/212852-PA (2022).

[49] Bazyari, A., Soulgani, B. S., Jamialahmadi, M., DehghanMonfared, A. & Zeinijahromi, A. Performance of smart water in clay-rich sandstones: Experimental and theoretical analysis. *Energy Fuels* **32**, 10354–10366. 10.1021/acs.energyfuels.8b01663 (2018). 10.1021/acs.energyfuels.8b01663

[50] Lashkarbolooki, M., Ayatollahi, S. & Riazi, M. The impacts of aqueous ions on interfacial tension and wettability of an asphaltenic–acidic crude oil reservoir during smart water injection. *J. Chem. Eng. Data* **59**, 3624–3634 (2014). 10.1021/je500730e

