# Supplementary material for: Theoretical and experimental investigation of the impact of oil functional groups on the performance of smart water in clay-rich sandstones
Source: Sci Rep. 2024 Aug 30;14:20172. doi: 10.1038/s41598-024-71237-1 (PMC11364868; doi:10.1038/s41598-024-71237-1)
Supplement: Supplementary file 1 — Supplementary Information. [file 41598_2024_71237_MOESM1_ESM.docx]

Supplementary

The values for zeta potential (mv) measured in this study

| **No** | **Aqueous Phase** | **Kaolinite** | **Quartz** | **Oil (A)** | **Oil (B)** |
| --- | --- | --- | --- | --- | --- |
| 1 | FW | 0 | 0 | 0 | 0 |
| 2 | DW | -49 | -32 | -20 | -4 |
| 3 | Na10LSW | -42 | -23 | -12 | -2 |
| 4 | Ca10LSW | -17 | -11 | -9 | -3 |
| 5 | Na50LSW | -26 | -15 | -12 | -4 |
| 6 | Ca50LSW | -9 | -5 | -6 | -1 |
